# Urban and Rural Patients Perspectives on Late Diagnosis of Breast Cancer in Tanzania: A Qualitative Study

**DOI:** 10.1200/GO.22.67000

**Published:** 2022-05-05

**Authors:** Elizabeth F. Msoka, Perry M. Cyril, Furaha Serventi, Brenda C. Kitali, Vivian F. Saria, Gilleard G. Masenga, Blandina T. Mmbaga, Lily Gutnik

**Affiliations:** ^1^ Kilimanjaro Clinical Research Institute, Moshi, Tanzania; ^2^ Kilimanjaro Christian Medical Centre, Moshi, Tanzania; ^3^ Kilimanjaro Christian Medical Centre, Kilimanjaro Christian Medical University College, Moshi, Tanzania; ^4^ The University of Alabama at Birmingham, Birmingham, AL

## Abstract

In Tanzania, women are diagnosed at an advanced stages of breast cancer. The aim of this study is to identify reasons for this finding from the patient perspective.

**METHODS:**

Semi-structured in-depth interviews were conducted in inpatient and outpatient settings in the Kilimanjaro Cancer Care Center. Thematic coding via grounded theory technique was done by two independent reviewers.

**RESULTS:**

Ten 10 patients (five rural and five urban) participated in the study. The average ages was 48. Seven (70.0%) were Christian. Eight (80.0%) patients had primary education, six (60.0%) were married, and eight (80.0%) were not employed. Two women (20.0%) had stage 2, four women (40.0%) had stage 3, and 4 (40.0%) had stage 4. The most common reasons reported by patients for advanced stage breast cancer at time of diagnosis are initially seeking care from traditional healers (n = 6, 60.0%), lack of breast cancer sign and symptoms knowledge (n = 8, 80.0%), misconceptions about breast cancer treatment especially mastectomy (n = 5, 50.0%), distrust of conventional medicine (n = 4, 40.0, seeking support from the religious leaders (n = 6, 60.0%). Furthermore, we found that, almost all (n = 9, 90.0%) of the patients express their concern regarding financial challenges to access care and treatment for cancer. For example (n = 3, 30.0%) said payment for cancer treatment is too costly and six patients (60.0%) stated that lack of money for transportation.

**CONCLUSION:**

Lack of adequate breast cancer knowledge and awareness, patient provider relationships, and access to care are the most common mentioned reasons for advanced disease at time of diagnose among breast cancer patients. Engagement with the community through cultural sensitive public health campaigns and interventions must be designed to alleviate the misconceptions and knowledge gap in these communities. System level change is needed to improve access to care.

