# Erratum

**DOI:** 10.1200/GO.22.00185

**Published:** 2022-06-17

The meeting abstract by Msoka et al entitled “Urban and Rural Patients Perspectives on Late Diagnosis of Breast Cancer in Tanzania: A Qualitative Study” (*JCO Glob Oncol* 10.1200/GO.22.67000) was published online May 5, 2022, with an error.

In the author list, Elizabeth Msoka, MSc^1,2^ should have been Elizabeth Msoka, MSc^1,2**,3**^

This has been corrected as of June 17, 2022. The authors apologize for the error.

